# Cost-effectiveness analysis of a communication-focused therapy for pre-school children with autism: results from a randomised controlled trial

**DOI:** 10.1186/s12888-015-0700-x

**Published:** 2015-12-21

**Authors:** Sarah Byford, Maria Cary, Barbara Barrett, Catherine R. Aldred, Tony Charman, Patricia Howlin, Kristelle Hudry, Kathy Leadbitter, Ann Le Couteur, Helen McConachie, Andrew Pickles, Vicky Slonims, Kathryn J. Temple, Jonathan Green, the PACT Consortium

**Affiliations:** King’s Health Economics, Box P024, King’s College London, De Crespigny Park, London, SE5 8AF UK; Stockport NHS Foundation Trust, Stockport, SK2 7JE UK; Department of Psychology, Box P077, King’s College London, De Crespigny Park, London, SE5 8AF UK; Olga Tennison Autism Research Centre, School of Psychology and Public Health, La Trobe University, Melbourne, Australia; Institute for Brain, Behaviour and Mental Health, University of Manchester, Oxford Road, Manchester, M13 9PL UK; Institute of Health and Society, Newcastle University, Sir James Spence Institute, Royal Victoria Infirmary, Queen Victoria Road, Newcastle upon Tyne, NE1 4LP UK; Biostatistics, Box P020, King`s College London, De Crespigny Park, London, SE5 8AF UK; Evelina Children`s Hospital, King`s College London, London, SE1 7EH UK; Manchester Academic Health Sciences Centre, Jean McFarlane Building, Oxford Road, Manchester, M13 9PL UK

**Keywords:** Autism, Economic evaluation, Cost-effectiveness, Pre-school, Communication

## Abstract

**Background:**

Autism is associated with impairments that have life-time consequences for diagnosed individuals and a substantial impact on families. There is growing interest in early interventions for children with autism, yet despite the substantial economic burden, there is little evidence of the cost-effectiveness of such interventions with which to support resource allocation decisions. This study reports an economic evaluation of a parent-mediated, communication-focused therapy carried out within the Pre-School Autism Communication Trial (PACT).

**Methods:**

152 pre-school children with autism were randomly assigned to treatment as usual (TAU) or PACT + TAU. Primary outcome was severity of autism symptoms at 13-month follow-up. Economic data included health, education and social services, childcare, parental productivity losses and informal care.

**Results:**

Clinically meaningful symptom improvement was evident for 53 % of PACT + TAU versus 41 % of TAU (odds ratio 1.91, *p* = 0.074). Service costs were significantly higher for PACT + TAU (mean difference £4,489, *p* < 0.001), but the difference in societal costs was smaller and non-significant (mean difference £1,385, *p* = 0.788) due to lower informal care rates for PACT + TAU.

**Conclusions:**

Improvements in outcome generated by PACT come at a cost. Although this cost is lower when burden on parents is included, the cost and effectiveness results presented do not support the cost-effectiveness of PACT + TAU compared to TAU alone.

**Trial registration:**

Current Controlled Trials ISRCTN58133827

**Electronic supplementary material:**

The online version of this article (doi:10.1186/s12888-015-0700-x) contains supplementary material, which is available to authorized users.

## Background

Autism is a severe neurodevelopmental disability associated with impairments that have life-time consequences for the health and quality of life of diagnosed individuals [1] and a substantial impact on families [2]. The annual cost of supporting children with autism spectrum disorders in the UK has been estimated at between £3.1 and £3.4 billion, dependent on the estimated proportion with intellectual disability. Almost 50 % of the estimated costs were accounted for by special education, including early intervention services, with parental productivity losses and respite care accounting for approximately 15 % each. Medical services accounted for only 10 % of the total [2].

There is growing interest in early interventions for children with autism, [3, 4], yet despite the substantial economic burden, there is little evidence of the cost-effectiveness of such interventions with which to support resource allocation decisions [5, 6]. Without such evidence, it is impossible to determine whether the resources currently spent on interventions for children with autism represent good value for money, or whether greater benefits for children could be generated by spending the money in other ways.

A number of cost-savings analyses of early intensive behavioural interventions (EIBI) for children with autism have been undertaken, which generally suggest initial expenditure on EIBI would be more than offset by subsequent cost-savings over the long-term, primarily in education and health care [7–10]. However, no full economic evaluations, involving a formal assessment of both costs and patient benefits in a comparative analysis of two or more alternative interventions for children with autism have been conducted.

This paper reports the results of an economic evaluation of a pre-school intervention for children with autism carried out within a multi-centre, randomised controlled trial – the Pre-school Autism Communication Trial (PACT) [11]. The CONSORT 2010 randomised trial checklist for the PACT trial is reported in Additional File 1. 

## Methods

### Hypothesis

The PACT trial compared the PACT intervention plus treatment as usual (TAU) with TAU alone for pre-school children with autism [11]. The economic hypothesis was that the additional costs of PACT would be offset by improvements in patient outcomes and/or savings in the use of other services, compared with TAU alone.

### Trial design and participants

Families with a child aged 2 years to 4 years 11 months meeting criteria for autism were recruited to a parallel randomised controlled trial with one-to-one allocation from specialist centres in London, Manchester and Newcastle, UK, between September 2006 and February 2008. Autism was defined according to international standard diagnostic tests, which include meeting criteria on the social and communication domains and algorithm total of the Autism Diagnostic Observation Schedule-Generic (ADOS-G) [12] and on two of three algorithm domains of the Autism Diagnostic Interview Revised [13]. Exclusion criteria included children with a twin with autism; a nonverbal age equivalent to 12 months or younger on the Mullen Early Learning Scales [14]; epilepsy requiring medication; severe hearing or visual impairment in a parent or the child; or a parent with a severe psychiatric disorder requiring treatment.

### Randomisation and masking

After consent was obtained and baseline assessments were carried out by trial research assessors, the PACT manager allocated a sequential identification number and provided a statistician at the independent Christie Clinical Trials Unit in Manchester with the child’s number, treatment centre, age and autism severity. This statistician ran an allocation schedule that was computer-generated by use of probabilistic minimisation of imbalance in the marginal distribution of treatment centre, age (≤42 months or >42 months), and autism severity (ADOS-G algorithm score 12–17 or 18–24). The statistician then telephoned the treatment allocation to the trial manager, who informed clinical sites. Research assessors and supervising staff were blind to treatment allocation but allocation could not be masked from therapists or families. The study was approved by the Manchester Multicentre Research Ethics Committee and was conducted in accordance with the ethical standards defined in the 1964 Declaration of Helsinki and its later amendments; at least one parent of each child provided written consent.

### Interventions

The PACT therapy was a developmental oriented, parent-directed and video-aided intervention that was moderated and matched to parental style. The intervention targeted social interactive and communication impairments in autism and was delivered by specially trained speech and language therapists [11]. The trial had six treatment therapists, two at each site and 3 lead therapists, one at each site. The lead therapist provided weekly supervision on case management and setting appropriate targets for treatment therapists.

The rationale of the intervention was that children would respond with enhanced communicative and social development to a style of parent communication adapted to their impairments. The aim was to increase parental sensitivity and responsiveness to child communication and reduce mistimed parental responses by working with the parent and using video-feedback methods to address parent–child interaction. Further incremental development of the child’s communication was helped by promotion of a range of strategies such as action routines, familiar repetitive language, and pauses.

The intervention consisted of an assessment session followed by fortnightly one-to-one clinic sessions for six months. Each session lasted approximately two and a half hours and was conducted between therapist and parent with the child present. This was followed by monthly booster sessions for six months (up to a maximum 19 sessions including the assessment session). Between sessions, families were asked to do 30 min of daily home practice. Clinic sessions were videotaped and 44 of these (with 37 participants), were selected by stratified randomisation to balance therapist and treatment stage, in order to assess treatment fidelity. Videos were double-coded for therapist fidelity against 14 criteria by PH, ALC and JG. Fidelity was demonstrated with a median of 13.4 criteria being met (inter-quartile range 12.5 to 14.0) per session.

The intervention was manualised and staged to represent the typical developmental progression of prelinguistic and early language skills. Stages were as follows: Stage 1 Establishing shared attention; Stage 2 Synchronicity and sensitivity; Stage 3 Focusing on language input; Stage 4 Establishing routines and anticipation; Stage 5 Increasing communication functions; Stage 6 Expanding language and conversations. Each stage had a specific aim and strategies that the therapist targeted with the parent. The intervention always started at Stage 1 with at least 2–3 sessions focussing on this stage. Subsequent progression was determined by the child’s developmental readiness and the pace of the parent in working through the goals. The intervention had measurable criteria for moving from one stage to the next that enabled the therapist to judge if the parent and child had accomplished a level of skill at that particular stage. Children remained at different stages for different periods of time and not all children accomplished the higher stages 5 or 6.

Full details of the background to the PACT intervention, and the development, principles, aims, trial procedure and stages of the PACT intervention can be found in the web appendix to the PACT clinical paper. For more on the PACT trial protocol, please see www.bbmh.manchester.ac.uk/pact.

Families in both groups continued with TAU provided by local services, which commonly includes paediatricians and speech and language therapists, alongside a variety of other health, social care and education-based services [6].

### Clinical outcomes

Outcomes were assessed at baseline, 7 and 13-months after randomisation. The primary outcome was severity of autism symptoms measured using the ADOS-G social communication algorithm score [12], researcher assessed in interview with parents and modified to improve sensitivity to change in children with no spoken words at baseline [11]. The ADOS-G has cut-off points for autism and autism spectrum disorder. Higher scores indicate greater severity. Secondary measures included video-rated, parent–child interaction during naturalistic play (proportion of parental responses that were synchronous, proportion of child communication with the parent that were initiations and proportion of time spent in mutual shared attention) [11], child language and social communication using the researcher assessed Preschool Language Scales [15], and adaptive functioning in school using the Vineland Adaptive Behaviour Scales, Teacher Rating Form [16], rated at endpoint by face-to-face interview with teachers in nurseries, reception class, or other appropriate carer who was not a member of the family. Full details of the primary and secondary outcomes measured in the PACT trial can be found in the previously published clinical paper [11].

### Resource use and cost

The economic evaluation took two cost perspectives: 1) a service perspective of particular interest to public sector policy-makers, including all hospital, community and school-based health, social and education services, and 2) a societal perspective to capture the full economic implications, which additionally included schooling and childcare costs, parental out-of-pocket expenditure (aids and adaptations to the home, training courses etc.), productivity losses (time off work due to child’s autism) and informal (unpaid) care. For informal care, parents were asked to estimate the additional hours of care they provide to their child on an average day over and above what they would provide for a child without autism, separated into the following categories: personal care, transport, housework and shopping, interacting/socialising/playing and night-time supervision.

Data on all the service use and other resource use associated with these two perspectives were collected using the Child and Adolescent Service Use Schedule (CA-SUS) and the Carer Service Use Schedule (CARER-SUS), designed in previous research with young people [17, 18]. Both schedules were adapted for the purpose of this study using expert opinion and pilot testing [6]. Data were collected in interview with parents at baseline (covering the previous six months) and 7 and 13-month follow-up (covering the period since last interview). Data on use of the PACT intervention were collected from therapist records. Details of other (non-PACT) speech and language therapy were collected by trial therapists from NHS speech and language therapists.

All resources used were then costed by applying unit costs, in UK pounds sterling, for the financial year 2006–2007. Intervention sessions were costed on the basis of the salary of the PACT therapists and any professional assistance required. A cost per-hour was calculated including employer costs (National Insurance and superannuation contributions), overheads (capital, administrative and managerial) [19] and supervisor costs. Indirect time (supervision, training, preparation, etc.) was estimated using information provided by trial therapists on the ratio of time spent in face-to-face contact to time spent on other activities. Travel costs to home visits were included.

Other unit costs were obtained from published sources [19–21], national surveys [22], mainstream retailers of non-prescription drugs, and government departments for school costs. Productivity losses were calculated using the human capital approach, which involves multiplying time off work due to the child’s illness by the parent’s salary [23]. Informal care costs were calculated using the market price approach, which applies the price that would be paid if the care were provided by a formal caregiver [24], in this instance the cost of a home care worker was used [19].

### Statistical analysis

PACT was powered on the basis of a clinically meaningful improvement in ADOS-G score between baseline and follow up of ≥4 points; modelled as equivalent to a 7 month increase in age-equivalent adaptive functioning [11]. Target recruitment was 144 families, and was calculated on the basis of a pilot study effect size for ADOS-G of 0 · 92 [25] and allowing for 10 % attrition, to provide greater than 99 %, 98 %, 90 %, and 75 % power for effect sizes of 0 · 8 SD (reduction of about 4 · 0 points), 0 · 6 SD (reduction of about 3 · 0 points), 0 · 5 SD (reduction of about 2 · 5 points), and 0 · 4 SD (reduction of about 2 · 0 points), respectively, with a two-sided *p* value of 0 · 05.

All economic analyses were carried out on an intention-to-treat basis using an analysis plan finalised prior to data analysis. Statistical tests were applied to cost differences, but not differences in resource use, to avoid the risk of finding significant differences by chance, as a result of multiple-significance testing. Although cost data are often skewed, as a result of small numbers of high cost individuals, analyses compared mean costs using standard t-tests to enable inferences to be made about the arithmetic mean, which is a more meaningful statistic for budgetary and planning purposes than the median [26]. The validity of applying parametric tests was confirmed using non-parametric bootstrapping [27], as recommended for the analysis of cost data [26]. Multiple regression was used to adjust for pre-specified baseline characteristics: gender, age, centre, autism severity (ADOS-G modified algorithm score), costs, parental occupation (at least one parent with a professional or administrative occupation versus other), parental education (at least one parent with post-16 qualifications versus other) and ethnicity (both parents white versus other).

Full economic data were available for 94.1 % of the sample (n = 143). Full follow-up data were missing for only five families (3.3 %), so multiple imputation was not considered necessary. However, the impact of missing data was explored for the four families (2.6 %) with partial data (7-month data available but not 13-month), using the last value carried forward. This is in line with evidence to suggest that past service use is a significant predictor of future costs [18, 28].

The method of economic evaluation applied was cost-effectiveness analysis, which is the most commonly adopted approach to economic evaluation in health care and involves the valuation of effects in a single disease-specific outcome measure. The outcomes of two or more interventions are then combined with their respective costs to provide a measure of relative cost-effectiveness that can be compared to other interventions employing the same measure of effect. In the current study, cost-effectiveness was explored in terms of the primary outcome measure (ADOS-G) using the net benefit approach, a framework for the analysis of uncertainty in cost-effectiveness analysis [29]. Since PACT was powered on the basis of a clinically meaningful improvement in ADOS-G score between baseline and follow up of ≥4 points, the economic evaluation thus assessed effectiveness in terms of the proportion of children demonstrating this level of ADOS-G improvement.

A joint distribution of incremental mean costs and effects for the two groups was generated using non-parametric bootstrapping [27]. These data were used to explore the probability that each treatment is the optimal choice, subject to a range of possible maximum values (ceiling ratio) that a decision-maker might be willing to pay for a 1 % increase in the proportion of patients achieving a clinically meaningful ADOS-G improvement. Cost-effectiveness acceptability curves (CEAC) were generated by plotting these probabilities for a range of possible values of the ceiling ratio [30, 31]. CEACs are a recommended decision-making approach to dealing with uncertainty around the estimates of expected costs and effects and uncertainty regarding the maximum cost-effectiveness ratio a decision-maker would consider acceptable [32, 33].

## Results

### Participants

**Fig. 1:**
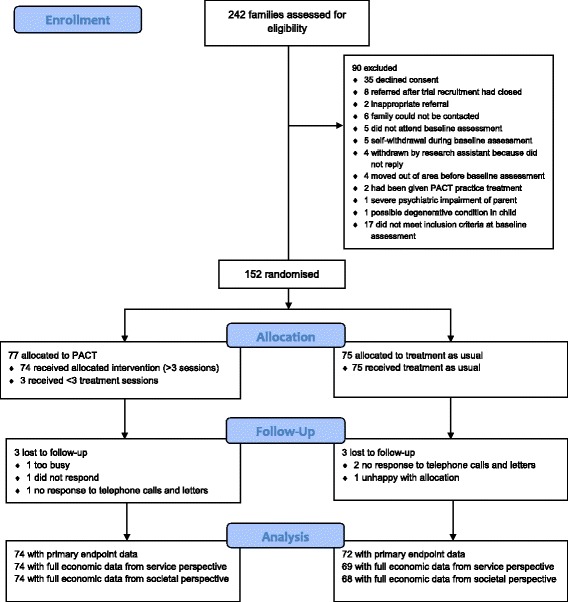
CONSORT flow diagram

Figure 1 shows the CONSORT diagram for the trial. 152 children were randomised to PACT + TAU (n = 77) or TAU (n = 75). Loss to follow-up was low (n = 6; 4 %) and primary outcome data at final follow-up were available for 96 % of the sample (n = 74 PACT + TAU; n = 72 TAU). Full data for the economic evaluation taking the service perspective were available for 94 % of the sample (n = 74 PACT + TAU; n = 69 TAU) and taking the societal perspective were available for 93 % (n = 74 PACT + TAU; n = 68 TAU). A comparison of baseline characteristics between those included and those missing revealed no significant differences. There were no significant differences in baseline characteristics between the two groups, apart from parental education, with the PACT + TAU families being more likely to have at least one parent with post-16 qualifications (Table 1). Participants were recruited between September 2006 and February 2008; assessments at the 13-month endpoint were carried out between September 2007 and March 2009.

**Table 1:** Baseline characteristics

	PACT + TAU	TAU
	(*n* = 74)	(*n* = 69)
Gender (female), n (%)	6 (8)	7 (10)
Age (years), median (range)	4 (2 to 5)	4 (2 to 5)
Study centre, n (%)		
London	24 (32.43)	26 (37.68)
Manchester	26 (35.14)	22 (31.88)
Newcastle	24 (32.43)	21 (30.43)
Parental qualifications (at least one parent post 16), n (%)*	63 (85)	43 (62)
Parental occupation (professional/administrative), n (%)	49 (66)	42 (61)
Ethnicity (both parents white), n (%)	45 (61)	38 (55)
ADOS-G algorithm score, mean (s.d.)	19.56 (4.35)	19.38 (4.11)
Proportion of parent communications with the child that were synchronous, mean (s.d)	29.32 (12.48)	27.21 (11.18)
Total health, education and social service cost in previous six months, mean £ (s.d.)	1422 (1035)	1366 (822)

*p = 0.002

### Clinical outcomes

At the 13-month endpoint, autism symptoms were reduced by 3.9 points (SD 4.7) on the ADOS-G in the PACT + TAU group and by 2.9 points (SD 3.9) in the TAU group, representing a between-group effect size of −0.24 (95 % CI −0.59 to 0.11). A clinically meaningful improvement was in evidence for 53 % of the PACT + TAU group compared to 41 % for TAU (odds ratio 1.91, 95 % CI 0.94 to 3.87, *p* = 0.074). Treatment effect was strong for parental synchronous response to child (between group effect size 1.22, 95 % CI 0.85 to 1.59) and moderate for child communication initiations with parent (between group effect size 0.41, 95 % CI 0.08 to 0.74).

### Resource use

Resource use over 13-months is detailed in Table 2. The cohort accessed a wide range of health, education, social care and childcare services and attended a range of schools and child care facilities (including nurseries, playgroups, childminders, holiday clubs and home tutors). Few differences were observed between the two groups.

**Table 2:** Resource use per participant during the 13-month follow-up period

	PACT + TAU	TAU
	(*n* = 74)	(*n* = 69)
	Mean (s.d.)	Mean (s.d.)
Speech and language therapy:		
PACT sessions	15.57 (4.37)	0.00 (0.00)
NHS speech and language therapy sessions	14.20 (15.45)	12.74 (15.28)
Other community health, education and social services:		
General practitioner contacts	2.69 (2.86)	2.51 (3.26)
General practice nurse contacts	0.91 (2.05)	1.03 (1.94)
Health visitor contacts	0.84 (2.06)	0.88 (1.59)
Community paediatrician contacts	0.92 (1.04)	0.84 (1.08)
Clinical psychologist contacts	0.18 (0.56)	0.87 (3.58)
Social worker contacts	1.04 (2.24)	0.71 (1.69)
Occupational therapist contacts	1.58 (9.99)	0.78 (2.20)
Physiotherapist contacts	0.11 (0.39)	0.10 (0.46)
Dietician contacts	0.22 (0.50)	0.16 (0.53)
Educational psychologist contacts	0.38 (0.79)	0.32 (0.72)
Special Education Needs Coordinator contacts	0.64 (2.93)	0.58 (3.74)
Portage worker contacts	1.34 (5.05)	1.87 (6.20)
Play worker contacts	0.31 (2.45)	1.91 (9.24)
Nutritionist contacts	0.03 (0.16)	0.16 (0.98)
Homeopath contacts	0.00 (0.00)	0.04 (0.36)
Osteopath contacts	0.96 (4.44)	0.32 (1.87)
Art worker contacts	0.70 (6.04)	0.00 (0.00)
Voluntary sector service contacts	4.57 (12.32)	4.19 (10.23)
Voluntary sector telephone helpline calls	1.65 (4.26)	1.06 (2.88)
Other community service contacts^a^	9.39 (35.31)	2.51 (6.94)
Hospital-based health services:		
Hospital nights	0.09 (0.38)	0.35 (1.80)
Outpatient visits	2.38 (3.94)	1.88 (3.92)
Accident and emergency visits	0.46 (0.80)	0.39 (1.00)
Education and childcare:		
Mainstream nursery weeks	25.78 (24.67)	20.42 (21.16)
Specialist nursery weeks	6.77 (16.09)	11.13 (20.74)
Mainstream playgroup weeks	3.61 (9.33)	3.00 (14.06)
Specialist playgroup weeks	5.09 (13.02)	7.14 (16.78)
Mainstream school weeks	13.07 (19.59)	16.59 (23.29)
Specialist school weeks	8.95 (17.67)	8.16 (18.95)
Holiday club weeks	0.39 (3.37)	0.19 (1.45)
Live in childcare weeks	0.16 (1.39)	0.00 (0.00)
Home tutor weeks	1.19 (6.78)	0.00 (0.00)
Childminder weeks	3.58 (13.24)	3.38 (10.84)
Parental productivity losses and informal care:		
Productivity loss total hours	45.07 (76.36)	25.47 (57.89)
Informal care hours per day	11.23 (7.18)	12.44 (7.00)

^a^Includes dentist, community autism specialist, family support worker, home care worker, audiology, podiatrist, ophthalmology, walk-in centre, podiatrist

Total productivity losses were low, although larger in the PACT + TAU group (45 versus 26 h in total over 13 months). Informal care hours were high in both groups but lower for PACT + TAU (11.23 versus 12.44 h per day).

### Service costs

Total service costs per participant over the 13-month follow-up are detailed in Table 3. The average cost of a PACT session was estimated to be £264 and the total cost of the PACT intervention was £4,105 per child, on average; the mean number of sessions attended was 16 out of a maximum of 19.

**Table 3:** Total cost of services per participant over the 13-month follow-up period (£), including sensitivity analysis

	PACT + TAU		TAU			
	*n*	Mean (s.d.)	*n*	Mean (s.d)	Difference (95 % CI)	*p*-value^*^
PACT	74	4105 (2122)	69	0 (0)	4105 (3599 to 4610)	0.000
NHS speech and language therapy	74	568 (618)	69	510 (611)	59 (−145 to 262)	0.555
Other community health, education and social services	74	1200 (2026)	69	1000 (1187)	200 (−355 to 754)	0.612
Medication	74	321 (1646)	69	110 (422)	210 (−193to 614)	0.434
Hospital-based health services	74	392 (651)	69	430 (930)	−38 (−302 to 226)	0.613
Total health, education and social services	74	6539 (3378)	69	2050 (1633)	4489 (3602 to 5377)	0.000
Last observation carried forward for missing cases	74	6539 (3378)	73	2213 (1850)	4326 (3436 to 5215)	0.000

*Adjusted for gender, age, centre, baseline ADOS-G score, baseline total costs, parental occupation, parental education and ethnicity

Total health, education and social service costs were £6,539 per child in the PACT + TAU group compared with £2050 in TAU (mean difference £4,489, 95 % CI £3,602 to £5,377; p < 0.001). The cost of the PACT intervention accounted for the majority of the observed cost difference. Analysis of missing data did not alter the findings.

### Societal costs

Broader societal costs, including the cost of schooling and childcare and parental costs (out-of-pocket expenditure, productivity losses and informal care), are reported in Table 4. Lower informal care costs in the PACT + TAU group reduced the cost difference between the groups to a non-significant amount (mean difference £1,385; 95 % CI –£8,468 to £11,239).

**Table 4:** Total societal cost per participant over the 13-month follow-up period (£)

	PACT + TAU	TAU	Difference (95 % CI)	*p*-value^*^
	(*n* = 74)	(*n* = 68)		
	Mean (s.d.)	Mean (s.d.)		
Total health, education and social services	6539 (3378)	2050 (1633)	4489 (3534to 5377)	0.000
Education and childcare	3743 (1451)	3578 (1462)	199 (−417 to 1004)	0.366
Parental out-of-pocket expenditure	1146 (2079)	788 (1019)	358 (−189 to 906)	0.149
Parental productivity losses	484 (837)	338 (918)	146 (−144 to 437)	0.621
Parental informal care	46007 (28722)	49814 (28970)	−3808 (−13350 to 5735)	0.459
Total societal cost	57919 (30157)	56534 (29375)	1385 (−8468 to 11239)	0.788

*Adjusted for gender, age, centre, baseline ADOS-G score, baseline total costs, parental occupation, parental education and ethnicity

### Cost-effectiveness analysis

**Fig. 2:**
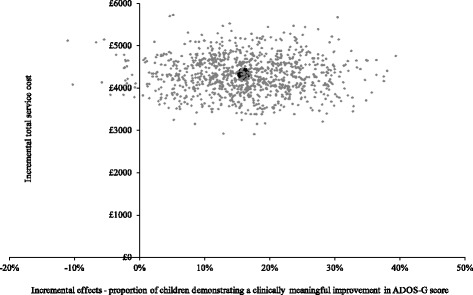
Cost-effectiveness plane using total service costs and ADOS-G score. Cost-effectiveness plane showing the bootstrapped, adjusted differences in total service costs and effects using the proportion of children demonstrating a clinically meaningful improvement in ADOS-G score

**Fig. 3:**
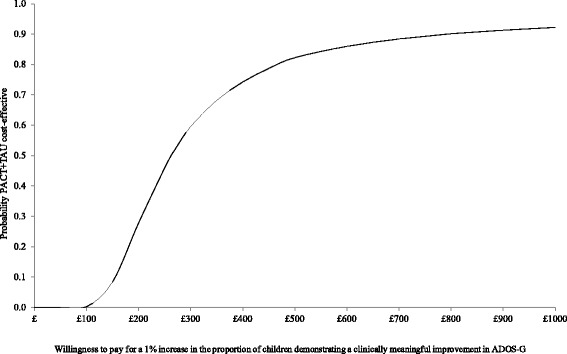
Cost-effectiveness acceptability curve using total service costs and ADOS-G score. Cost-effectiveness acceptability curve showing the probability that PACT + TAU is more cost-effective than TAU alone in terms of total service costs and proportion of children demonstrating a clinically meaningful improvement in ADOS-G score

In terms of service costs (health, social care and education), Fig. 2 shows the bootstrapped replications for incremental cost and effectiveness and demonstrates that PACT + TAU is more costly than TAU for all replications (points above the x-axis) and is associated with better outcomes for the majority of replications (points to the right of the y-axis). The CEAC in Fig. 3 illustrates the associated uncertainty. At low levels of willingness to pay per child for a unit improvement in the proportion of children who demonstrate a clinically meaningful ADOS-G improvement, there is a greater probability of TAU being the more cost-effective intervention. At willingness to pay levels of £265 and above, however, there is a greater probability of PACT + TAU being the more cost-effective option.

**Fig. 4:**
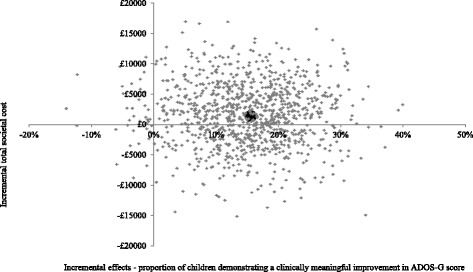
Cost-effectiveness plane using total societal costs and ADOS-G score. Cost effectiveness plane showing the bootstrapped, adjusted differences in total societal costs and effects using the proportion of children demonstrating a clinically meaningful improvement in ADOS-G score

**Fig. 5:**
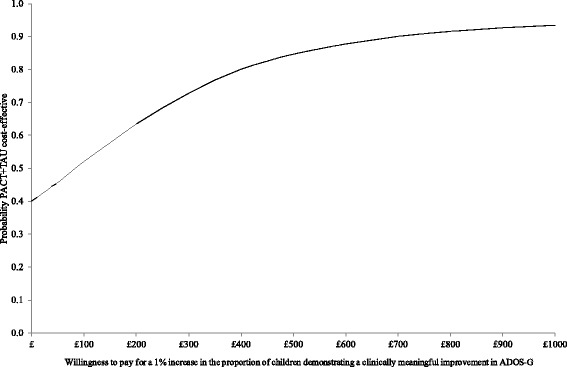
Cost-effectiveness acceptability curve using total societal costs and ADOS-G score. Cost-effectiveness acceptability curve showing the probability that PACT + TAU is more cost-effective than TAU alone in terms of total societal costs and proportion of children demonstrating a clinically meaningful improvement in ADOS-G score

In terms of societal costs, results were similar but more favourable for PACT + TAU due to reductions in the cost difference between the groups. Figure 4 demonstrates that PACT + TAU is more costly for approximately 60 % of the replications (compared to 100 % when considering only service costs) and is associated with better outcomes for the majority of replications. The CEAC in Fig. 5, shows PACT + TAU has a higher probability of being cost-effective than TAU if society is willing to pay £100 or more per child for a unit improvement in the proportion of children who demonstrate a clinically meaningful ADOS-G improvement.

Treatment effects in the PACT trial were positive for a number of targeted proximal outcomes, particularly parental synchronous responses to the child, also found to be positive in our pilot study [25]. This suggests the PACT therapy was more successful at improving parent–child social communication than reducing autism symptoms [11]. This finding was considered further in a secondary, exploratory cost-effectiveness analysis.

**Fig. 6:**
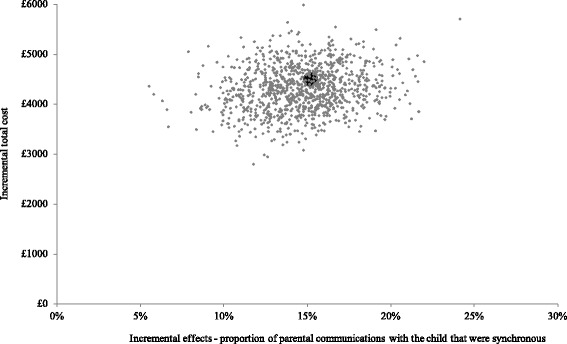
Cost-effectiveness plane using total service costs and parent synchrony. Cost effectiveness plane showing the bootstrapped, adjusted differences in total service costs and effects using the proportion of parent communications with the child that were synchronous

**Fig. 7:**
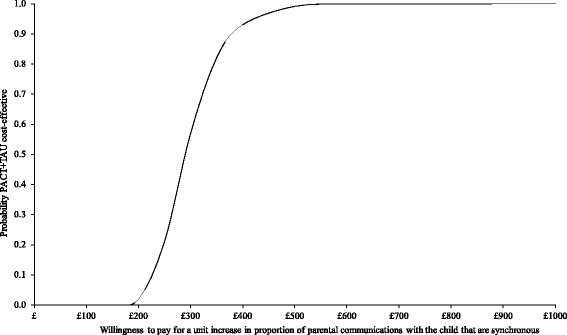
Cost-effectiveness acceptability curve using total service costs and parent synchrony. Cost-effectiveness acceptability curve showing the probability that PACT + TAU is more cost-effective than TAU alone in terms of total service costs and proportion of parent communications with the child that were synchronous

Results using parental synchronous responses and total service costs generated similar results to the primary outcome measure, as demonstrated in Figs. 6 and 7. The scatter-plot in Fig. 6 illustrates that PACT + TAU is more costly (above x-axis) and more effective (right of y-axis) than TAU for all replications. Figure 7 shows TAU having a higher probability of being cost-effective at low levels of willingness to pay but PACT + TAU being more likely to be cost-effective at willingness to pay of £300 or above per 1 % increase in the proportion of parental communications with the child that were synchronous.

## Discussion

The Pre-school Autism Communication Trial found a small group difference in autism symptoms in favour of the PACT intervention and concluded that it was not possible to recommend the addition of the PACT intervention to TAU for the reduction of autism symptoms [11]. However, this conclusion did not take into consideration cost or cost-effectiveness implications, without which it is not possible to determine whether the PACT intervention is an efficient use of resources.

### Resource use and cost

In terms of service use, few differences were observed between the two groups, with the exception of the PACT intervention, resulting in a statistically significant difference in total service cost that was driven by the cost of the PACT intervention. Thus the PACT intervention neither increased nor decreased participants’ use of other health, social or education services. This result contrasts with cost-savings demonstrated for EIBI in previous studies [7–10]. However, these studies all involved estimation of savings based on published evidence of the effectiveness of EIBI, and assumptions relating to the impact of EIBI on the need for special education, compared to no intervention. The relevance of these results to the UK is currently unknown and, as noted by Motiwala and colleagues [9], uncertainty surrounding the efficacy of EIBI suggests the need for further evaluation within a rigorous, randomised controlled design.

The inclusion of wider societal costs reduced the cost difference to a non-significant amount as a result of lower rates of informal care in the PACT + TAU group. However, confidence intervals were wide due to substantial variation in informal care hours, so these results should be interpreted cautiously. From a therapeutic point of view, there is some logic to the PACT + TAU group’s lower informal care estimates, since the PACT intervention aims to help parents feel more confident in recognising and meeting their child’s needs. Despite this, reported rates of informal care were large for both groups (over 11 h per day on average), highlighting the substantial burden placed on families of young children with autism. These rates are higher than in a previous study [34], which reported a mean of 8.6 h per day using similar methods. However, those findings were based on a sample of only nine children who were older than the PACT participants (mean age 7.8 versus 3.7 years).

Productivity losses were low in both groups. This may be because the majority of the children were in some form of education for some or all of the school day. However, these results exclude decisions taken by parents to stop working in order to support their child prior to the start of the trial, so the full extent of productivity losses is unknown.

Mean costs per child hide substantial variation, also evident at baseline [6]. Total service costs over 13-months ranged from £0 to £229 per week, whilst service costs plus education costs (all statutory provision) ranged from £41 to £322 per week. This suggests that after diagnosis, a proportion of young children with autism are failing to be provided with or failing to access available services and education facilities, perhaps because a need for additional support was not identified.

### Cost-effectiveness

Assessment of costs and outcomes independently suggests that PACT is associated with significantly greater costs and no significant difference in outcome, and thus should not be recommended as a cost-effective addition to TAU. Exploration of cost-effectiveness and the uncertainty associated with the cost and effectiveness data suggests that the addition of PACT to TAU would only have a higher probability of being cost-effective compared to TAU alone if society is willing to pay extra for improvements in outcome. In other words, adding the PACT intervention to TAU generates additional benefits compared to TAU alone, but at a price. Results were similar whether autism symptoms or parental synchrony are the measure of effect. These results are relevant to children with core autism and may not be generalizable to children with broad autism spectrum disorder.

### Limitations

There is no currently available societal value for a unit improvement in ADOS-G score, making it difficult to decide whether PACT should be recommended on the grounds of cost-effectiveness. The economists’ solution to such trade-offs (one intervention more effective, but more costly than another) is to use generic measures of health-related quality of life capable of generating such indicators as the quality adjusted life year (QALY) [35, 36]. These provide a common measure of output allowing comparisons to be made between diverse interventions and can be associated with societal values of willingness to pay to support decision making. The PACT trial was unable to include a generic outcome measure because, until recently, all available measures were developed for application to adult populations. Although some measures have now been adapted or developed for younger populations [37–39], these are recommended for children aged 7 years and over and thus are not suitable for a pre-school population. Nor have they been validated for use with children with autism, some of whom may be non-verbal. In the absence of a generic measure, it is up to policy-makers and service commissioners to decide whether the PACT intervention is worth paying for, given the levels of effectiveness generated. In addition, future studies should consider the inclusion of generic measures of quality of life for children and the use of proxy respondents.

This study is also limited by the retrospective, self-report nature of informal care, with evidence suggesting that self-reported informal care hours are over-inflated compared to prospective diaries [34]. This reduces the reliance we can have on the informal care data, which is notoriously difficult to measure accurately [40, 41]. In order to minimise reporting problems, responses were elicited from respondents in interview with the research assessors, allowing respondents the opportunity to ask questions if they were having difficulties completing the measure. Whilst there were still some problematic responses (for example, respondents reporting greater than 24 h a day), feedback from the interviewers provided a relatively good understanding of the difficulties (respondents feeling that they were supervising their children 24 h a day, alongside all other support provided), allowing the data to be adjusted to reduce the impact of this problem. Perhaps most importantly, however, these results highlight the need to explore this area in more detail and using more sophisticated methods, in order to confirm the findings.

## Conclusions

The addition of the PACT intervention to treatment as usual for children aged between 2 years and 4 years 11 months and meeting criteria for autism generated improvements in the severity of autism symptoms and in parental synchrony, but these improvements were not statistically significant, they required a substantial investment of health service resources and they did not generate cost-savings elsewhere in the healthcare system or in the education or social care systems. Although there was evidence of reductions in the burden on parents in the PACT group, which partly offset the cost of the PACT intervention, the cost and effectiveness results presented do not support the cost-effectiveness of PACT + TAU compared to TAU alone. Further research is needed to build a better understanding of the informal care implications of supporting young children autism and the validity of generic measures of outcome to better support resource allocation decisions in the future.

## Additional file

Additional file 1:
**CONSORT 2010 randomised trial checklist for the Pre-School Autism Communication Trial.** (DOC 219 kb)
